# Genes Required for Growth at High Hydrostatic Pressure in *Escherichia coli* K-12 Identified by Genome-Wide Screening

**DOI:** 10.1371/journal.pone.0073995

**Published:** 2013-09-11

**Authors:** S. Lucas Black, Angela Dawson, F. Bruce Ward, Rosalind J. Allen

**Affiliations:** 1 SUPA School of Physics and Astronomy, University of Edinburgh, Edinburgh, Midlothian, United Kingdom; 2 Institute of Cell Biology, School of Biological Sciences, University of Edinburgh, Edinburgh, Midlothian, United Kingdom; Georg-August-University of Göttingen Institute of Microbiology & Genetics, Germany

## Abstract

Despite the fact that much of the global microbial biosphere is believed to exist in high pressure environments, the effects of hydrostatic pressure on microbial physiology remain poorly understood. We use a genome-wide screening approach, combined with a novel high-throughput high-pressure cell culture method, to investigate the effects of hydrostatic pressure on microbial physiology *in vivo*. The Keio collection of single-gene deletion mutants in *Escherichia coli* K-12 was screened for growth at a range of pressures from 0.1 MPa to 60 MPa. This led to the identification of 6 genes, *rodZ, holC, priA, dnaT, dedD* and *tatC,* whose products were required for growth at 30 MPa and a further 3 genes, *tolB, rffT* and *iscS*, whose products were required for growth at 40 MPa. Our results support the view that the effects of pressure on cell physiology are pleiotropic, with DNA replication, cell division, the cytoskeleton and cell envelope physiology all being potential failure points for cell physiology during growth at elevated pressure.

## Introduction

Understanding the biophysical mechanisms that provide fundamental limits for life under extreme conditions has important implications for our ability to develop new biotechnological products, as well as for our understanding of the global biosphere [1] and, ultimately the potential for life on other planets. Hydrostatic pressure is one of the least well-understood environmental parameters in terms of its effects on cell physiology, even though high pressure deep-sea and subsurface environments constitute a large fraction of the Earth’s biosphere [2], [3], and their potential for pharmaceutical and biotechnological discoveries is increasingly being recognized [4]. While a significant body of work has focused on the factors limiting the survival of microorganisms exposed to killing pressures [3], [5], much remains to be learned about the physiological limits to growth at more moderate pressures, relevant to deep-sea or subsurface environments.

Hydrostatic pressure is known to have wide-ranging effects on biophysical processes [3]. Pressure in the range 10–40 MPa can cause dissociation of protein complexes, possibly due to closer packing of water molecules around the dissociated components [3], [6], [7], [8] (since Le Châtelier’s principle states that pressure shifts biochemical equilibria towards the state with the lower molecular volume). Other important biophysical effects of moderate pressure (10–40 MPa) include an increase in membrane lipid ordering [3], [9], which is compensated in deep sea organisms by an increase in the proportion of unsaturated fatty acids [10]. The proton-translocating ATPase is also known to be pressure sensitive [11]. Exposure to higher pressure, above 200 MPa, has been shown to cause protein unfolding [6], [12].

Here, we investigate the physiology of the bacterium *Escherichia coli* K-12 during growth at moderate hydrostatic pressure. *E. coli* is not a pressure-adapted organism, and is unlikely to have been subjected to elevated pressure during its recent evolutionary history. Thus, we do not expect it to show specific cellular adaptation mechanisms, as would be the case, for example, for temperature or acid stress. Nevertheless, *E. coli* has a remarkable ability to grow at pressures up to 50 MPa (approximately equal to the pressure at an ocean depth of 5 km); an ability that is shared by many other microbial species [13]. This is an extraordinarily high pressure tolerance compared to that of non pressure-adapted higher organisms. Understanding the biophysical factors that ultimately limit the ability of *E. coli* to grow at pressure can provide important insights into the fundamental limits of life in high-pressure environments.

Hydrostatic pressure has significant, and diverse, effects on the physiology of *E. coli*. Cell motility becomes diminished at pressures as low as 10 MPa, due both to inability to use existing flagella and inability to synthesize new flagella [14], [15], [16]. Despite increased lag times [17], [18], cell growth continues up to 50 MPa, but cell division becomes inhibited at ∼20 MPa, leading to filamented cells [18]. This filamentation may be related to inhibition of FtsZ ring formation, since filamented cells imaged after fixation at 50 MPa do not show FtsZ rings, and FtsZ filaments have been shown to dissociate *in vitro* at 40 MPa [19]. At 50 MPa, DNA synthesis becomes inhibited [20]: this is believed to be due to inhibition of DNA replication initiation or termination [20]. Protein synthesis is also inhibited at about 58 MPa [17], [20], which may be due to loss of stability of ribosome complexes [21], [22]. RNA synthesis, however, continues at higher pressures, up to about 77 MPa [18], [20], [23], [24]. Higher pressure, above about 100–200 MPa [25], is lethal to *E. coli.* The mechanisms leading to cell death at these pressures are of general interest in the hydrostatic pressure processing of foods [3], [5], since exposure to killing pressures can offer an alternative sterilization method for foods whose taste and texture is sensitive to heat sterilization.

Other important studies have focused on the gene regulatory response of *E. coli* to pressure. In classic early work, Welch *et al*. [17] found using proteomic analysis that both the heat-shock and cold-shock responses were transiently up-regulated (relative to the mean protein synthesis rate) following pressure upshift to 55 MPa. This suggests that the physiological effects of pressure may overlap with both of these stresses, which may explain why *E. coli* is pressure-tolerant despite not having evolved under high-pressure conditions. A subsequent DNA microarray analysis of *E. coli* under sustained growth at elevated pressure also found up-regulation of heat-shock and cold-shock genes, as well as changes in expression in hundreds of other genes at 30 MPa and 50 MPa relative to atmospheric pressure (0.1 MPa) [26]. Other work has revealed down-regulation of expression of the *ompC* and *ompF* outer-membrane protein-encoding genes during growth at pressures up to 30 MPa [27]. Transient exposure to much higher pressure (100–400 MPa) has been shown to produce heat-shock [28], oxidative stress [29] and SOS responses [30], [31].

Here, we use a complementary approach, genome-wide screening of the Keio collection of single gene deletion mutants [32], to identify genes whose products are required for growth of *E. coli* at pressures of 30 MPa and 40 MPa. Genome-wide screening of deletion mutant libraries provides an important tool for understanding cell physiology. For environmental stresses to which *E. coli* is evolutionarily adapted, such as temperature or pH, such screening allows the identification of specific adaptation mechanisms. Since *E. coli* is not evolutionarily adapted to pressure, we expect our screen rather to point to those physiological processes which are “failure points” for cell growth under pressure. High-throughput high-pressure cell culture involves considerable technical challenges. To our knowledge only two previous genome-wide screening studies have focused on cell physiology during growth at pressure. For the piezophilic bacterium *Photobacterium profundum*, Lauro *et al* identified a number of genes required for growth at 45 MPa, with functions including chromosome replication, metabolism, and ribosomal structure and biogenesis [33]. For the yeast *Saccharomyces cerevisiae*, Abe *et al* identified 71 genes required for growth at 25 MPa, with diverse physiological functions [34]. Both these studies point to pleiotropic effects of pressure on cell physiology – a picture that is supported by our results. Our study in *E. coli* should facilitate the identification of common patterns in the physiological response to pressure, across different organisms.

## Materials and Methods

### High-throughput Growth at Pressure

Genome-wide screening for growth at pressure is challenging because standard high-pressure cell culture methods typically allow cultivation of only a few samples at a time, whereas genome-wide studies require many mutants to be analyzed simultaneously. We have developed a method which allows high-throughput, quantitative screening of microbial growth at pressure using 96-well microplates. The challenge here lies in sealing the microplates such that the seals are flexible, to transmit pressure to the well contents, are strong enough not to break at pressure, and are transparent, to allow rapid quantification of microbial growth via measurement of absorbance at 600 nm (A_600 nm_) using a microplate reader [35]. In our experiments, flat-bottomed 96-well microplates (Greiner-Bio-One) were filled with growth medium and inoculated such that each well was completely full (382 µl). Taking care not to spill the well contents, a MicroAmp transparent adhesive-coated film (Applied Biosystems) was placed over the top of the microplate and a hand roller was used to secure the bond between plate and film. Excess trapped air was avoided by applying the film at an angle. After trimming excess film, a thick layer of Araldite fast-setting epoxy adhesive was applied around the edges of the plate to ensure a firm bond. After allowing the epoxy to set, the microplates were placed into a 3-litre water-filled, temperature-controlled, pressure vessel, which was sealed and pressurized using a hand pump. All growth experiments were carried out at 37°C. The absence of contamination between neighbouring wells in the plate was verified in control experiments both at atmospheric pressure and at 30 MPa.

### Measurement of Growth Yield

To measure the growth yield of the cultures, after 24 h growth at pressure, the pressure vessel was depressurized and absorbance at 600 nm (A_600 nm_) was measured using a BMG FluoStar Optima plate reader, without removing the microplate seals. The MicroAmp film undergoes a slow reaction with the growth medium that causes it to increase in opacity during the 24 h growth period. To control for this, wells containing growth medium only were included in every experimental run. The A_600 nm_ value of the culture in a given well was blanked against the average A_600 nm_ value for the medium-only wells, measured at the same time point and after the same history of pressurization. This measurement procedure was validated using a microplate containing a dilution series of growth medium containing crystal violet dye at known A_600 nm_ (Figure S1).

### Bacterial Strains, Plasmids and Growth Media

The Keio collection [32] consists of 3985 single-gene deletion mutants in *E. coli* K12 strain BW25113 (Δ*(araD-araB)567*, Δ*lacZ4787*(::rrnB-3), lambda^-^, *rph-1*, Δ*(rhaD-rhaB)568*, *hsdR514*). In each mutant, a single open reading frame is completely replaced by a kanamycin resistance cassette flanked by *frt* sites. In our experiments, these strains were grown in Luria-Bertani (LB) broth supplemented with 25 mM glucose. For initial growth direct from freezer stocks this medium was supplemented with 30 µg/ml kanamycin. For complementation experiments we used the ASKA clone collection [36]. This consists of 4327 ORFs from *E. coli* K-12 strain W3110, cloned under the control of IPTG-inducible promoter *P_T5-lac_* into the high copy number plasmid pCA24N, which carries a chloramphenicol resistance cassette. The version of the ASKA collection without N-terminal GFP tags was used in this work. In our complementation experiments, the LB-glucose growth medium was supplemented with 12.5 µg/ml chloramphenicol. IPTG was not found to be necessary for induction of gene expression from the ASKA plasmids (this is probably due to leakage from the promoter, combined with the high plasmid copy number). Indeed, induction with IPTG had either no effect or a negative effect on the growth of our complemented strains.

### Screening the Keio collection for Growth at Pressure

The Keio collection is contained in a set of 45 96-well microplates which were stored at −80°C. To obtain starter cultures for our screening experiment, each plate was thawed slightly and used to inoculate (using a 96-well replicator) a fresh 96-well plate whose wells contained 200 µl growth medium; this plate was incubated at 37°C overnight with shaking. The resulting cultures were then used to inoculate (again using a 96-well plate replicator) fresh microplates, each containing 382 µl growth medium per well, which were sealed and pressurized as described above. After 24 hours’ incubation at 30 MPa and 37°C, the plates were depressurized and their growth yield was measured as described above. The whole Keio collection was also screened for growth at 0.1 MPa (data not shown). The 8 mutants that failed to grow at 0.1 MPa under the micro-aerobic conditions in our plates were excluded from our subsequent analysis (these were *yniC, thyA, oxyR, setB, yfcX, yfbU, yehD* and *stfE*).

### Rescreening a Subset of Mutants at a Range of Pressures

A subset of 88 mutants was intensively re-screened for growth at pressures ranging from 10 MPa to 60 MPa. This subset contained the 82 mutants which had shown the lowest growth yields in our screen of the entire Keio collection at 30 MPa (eliminating any which failed to grow at atmospheric pressure; see Table S1). We also included 3 mutants (*envC*, *gpmI* and *hns*) which had previously been identified in a non-quantitative pressure screen in our lab (*hns* has also been identified in previous work [26]), and 3 other mutants: *rpoS* (which encodes the alternative sigma factor σ^s^, the master regulator of the general *E. coli* stress response), *fliF* (which is a key component of the basal body of the *E. coli* flagellum) and *recD* (which has been shown to be required for replication of plasmid DNA at pressure [37]). In fact we did not observe pressure sensitivity for any of these mutants (see Table S1). A master 96-well plate was assembled containing these 88 mutants of interest, as well as 4 wells containing the parent strain BW25113 and 4 medium-only wells. This master plate was stored at −80°C (after adding glycerol to all wells). For screening experiments, the master plate was used to inoculate overnight starter cultures, which were used to provide inocula for the pressure screening. Growth and screening experiments were carried out as described above, with the addition that at least 4 replica plates were used for each pressure screen, in order to allow statistical errors on the measured growth yields to be assessed. Comparison of the results for replicate plates showed a high level of reproducibility (Figure S2); the level of plate-to-plate variability in individual A_600 _z_nm_ values was 8% (average standard deviation/mean).

### Verification of Pressure-sensitive Mutants by Complementation

To confirm the link between genotype and pressure-sensitive phenotype for putative pressure-sensitive mutants, we used the ASKA plasmid collection [36] to introduce the deleted gene back into the Keio mutant strains identified in our screens as being pressure-sensitive at 30 MPa or 40 MPa. Plasmids from the ASKA collection were introduced into CaCl_2_-treated competent cells of the appropriate Keio strains, using heat-shock transformation with 2–5 µl plasmid DNA, followed by selection for transformants on LB-agar plates containing 12.5 µg/ml chloramphenicol. These transformants were then screened for growth at 30 MPa or 40 MPa (as appropriate) as described above.

### Microscopy

To assess the morphology of pressure-sensitive and complemented strains grown at 0.1 MPa, 30 MPa or 40 MPa, bacterial cultures were grown at the appropriate pressure in 96-well microplates, for 24 hours as described above, but with a higher starting density (1∶8 dilution of overnight starter cultures). Immediately after depressurization, 6 µl of culture was placed on a microscope slide and imaged on a Nikon Ti-U inverted microscope with a 100× 1.40 NA Plan Fluo oil-immersion objective, using bright-field illumination.

## Results

### COG Classification Correlates with Growth at Pressure

To determine whether mutants lacking in particular physiological functions were more likely to grow poorly at pressure, we carried out a global analysis of the entire Keio collection, testing for correlation between the COG category (Clusters of Orthologous Groups of proteins) of the deleted genes and the growth of the corresponding mutant at pressure. The ratio of growth yield at 30 MPa to yield at 0.1 MPa (A_600 nm_(30 MPa)/A_600 nm_(0.1 MPa)) was significantly lower in mutants lacking genes from the Information Storage and Processing and Poorly Characterized COG categories, compared to the complete mutant set (p = 0.017 and p<0.0001 respectively), and was significantly higher for mutants lacking genes in the Metabolism category (p<0.0001). The Information Storage and Processing category comprises translation, ribosome structure and biogenesis, transcription, and DNA replication, recombination and repair; our analysis thus suggests that these processes may be important in growth at pressure.

### Six Genes are Essential for Growth of *Escherichia coli* K-12 at 30 MPa

**Figure 1:**
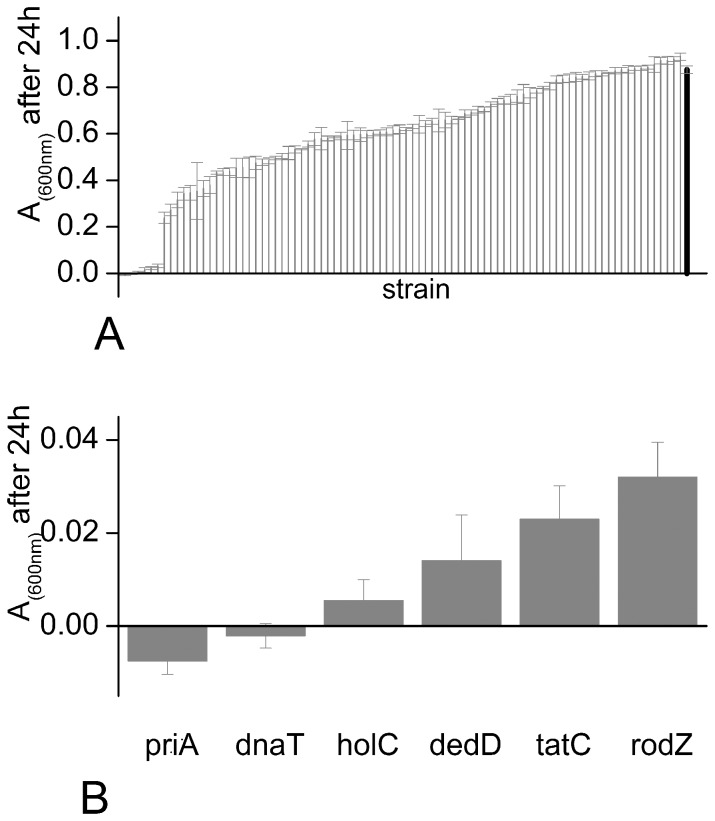
Six mutants fail to grow at 30 MPa. Panel A: Absorbance at 600 nm of cultures after 24 hours of growth at 30 MPa and 37°C, for the selected subset of mutants, arranged in ascending order. These results are also listed in Table S1. The results shown are averages of between 4 and 12 replicate experiments; error bars show the standard error in the mean (SEM) of the absorbance measurements. The A_(600 nm)_ value for the parent strain BW25113 is shown as the right-hand bar (black). Panel B: Zoomed-in view of Figure 6A, showing the A_(600 nm)_ values for the set of 6 mutants which failed to grow at 30 MPa. These mutants all showed significant growth at 0.1 MPa (see Table S1).

Quantitative screening of the entire Keio collection resulted in a collection of 82 “candidate” deletion mutants that showed the lowest growth yields at 30 MPa. Repeated rescreening of the candidate deletion mutants at 30 MPa revealed 6 mutants that consistently showed much poorer growth than the other strains (Figure 1 and Table S1). The genes lacking in these 6 mutants were *rodZ (*also known as *yfgA), holC, priA, dnaT, dedD* and *tatC*. Of these mutants, *priA* and *dnaT* showed no growth at all at 30 MPa (A_600 nm_(30 MPa)/A_600 nm_(0.1 Pa) = 0), *holC*, *dedD* and *tatC* showed virtually no growth (A_600 nm_(30 MPa)/A_600 nm_(0.1 Pa) = 0.01–0.03) and *rodZ* showed severely reduced growth (A_600 nm_(30 MPa)/A_600 nm_(0.1 Pa) = 0.06; see Table S1).

**Figure 2:**
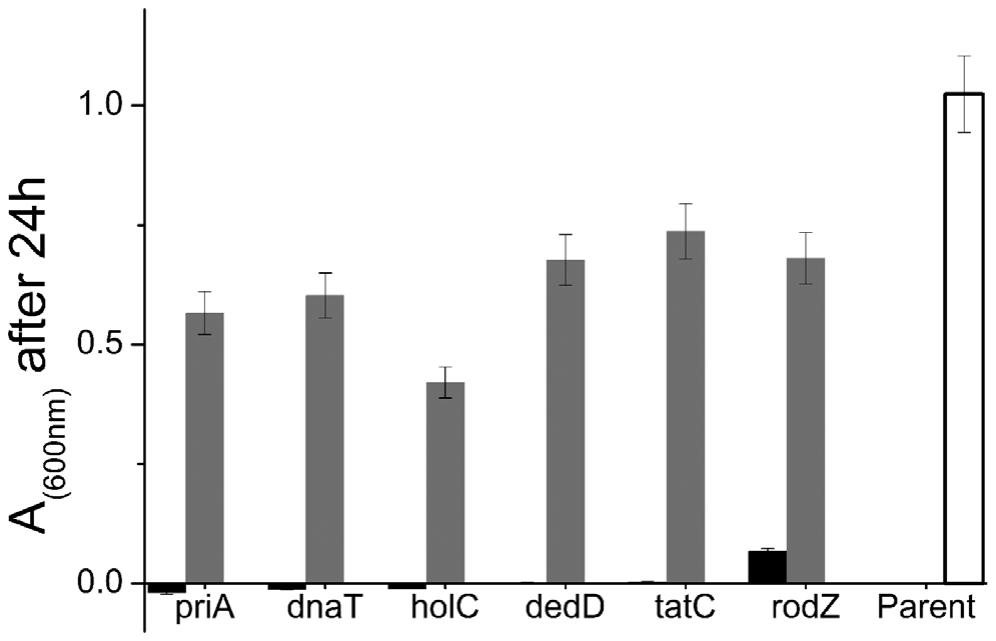
Complementation restores the ability of the six mutants to grow at 30 MPa. Absorbance at 600 nm of cultures after 24 hours of growth at 30 MPa and 37°C, for the 6 deletion mutant strains *rodZ, dnaT, holC, priA, dnaT, dedD* and *tatC* and for the corresponding complemented strains, as well as the parent strain BW25113. The results are the average of 8 replicate wells on the same plate, which show almost identical A_600_ values. The error bars are computed taking into account variability between replicate plates. From Figure S2, we find the average standard deviation in measured absorbance across replicate plates is 8% of the mean absorbance. We have used this value to compute the error bars in the figure.

The introduction of plasmids carrying the deleted gene back into these mutants (see Materials and Methods) resulted in almost complete restoration of the parent strain growth phenotype (Figure 2), confirming that the pressure-sensitive phenotype was indeed due to lack of the deleted gene.

**Figure 3:**
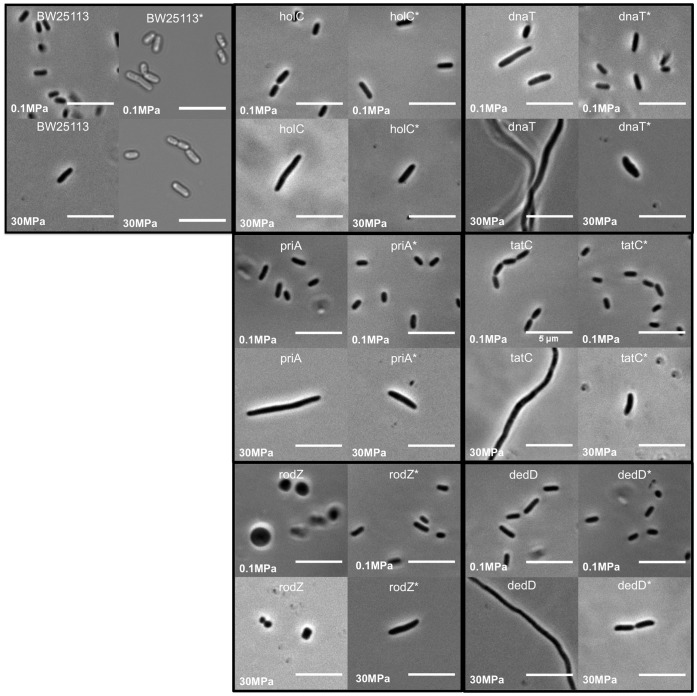
Cell morphology is affected in the pressure-sensitive mutants at 30 MPa. Phase contrast microscopy images of the parent strain BW25113 and the *dedD, dnaT, holC, priA, rodZ* and *tatC* deletion mutants, together with their corresponding complemented strains (denoted by asterisks), as well as BW25113 carrying the empty plasmid pCA24N (denoted by BW25113*), after 24 h at 0.1 MPa and 30 MPa. The upper images in each panel show cells grown at 0.1 MPa; the lower images show cells grown at 30 MPa. Left-hand images in each panel show the pressure sensitive mutant; right-hand images show the complemented strain. All scale bars are 10 µm.

Figure 3 shows the morphology of the 6 pressure-sensitive mutants at atmospheric pressure and after 24 h at 30 MPa (top and bottom left panels of each set). After 24 h at 30 MPa, the cells may of course be dead; nevertheless striking changes in morphology at pressure may give a clue as to the mechanism of growth inhibition/killing. The parent strain BW25113 showed a slightly elongated cell phenotype when grown at 30 MPa, with an average cell length of 2.7±0.1 µm (7 cells) versus 1.35±0.02 µm at 0.1 MPa (216 cells). This result is similar to observations for other *E. coli* K-12 strains [3], [24], [28]. The *holC* mutant appeared morphologically similar to the parent strain, with average cell length 4.4±0.1 µm at 30 MPa (8 cells) and 1.53±0.03 µm at 0.1 MPa (101 cells). In contrast, the *dnaT* mutant showed slightly elongated cells at 0.1 MPa (average length 3.3±0.5 µm; 45 cells) and filamented at 30 MPa (average cell length 13±2 µm; 19 cells); cells of the *priA* mutant also filamented at 30 MPa (average cell length 14±3 µm; 11 cells) although these appeared normal at 0.1 MPa (average cell length 1.3±0.3 µm; 112 cells). The *dedD* mutant likewise filamented at 30 MPa (average cell length 25.3±4.3 µm; 7 cells), but was only slightly elongated at 0.1 MPa (average length 2.3±0.1 µm; 145 cells). The *tatC* mutant showed a cell chaining phenotype at 0.1 MPa, as observed in other studies [39], and formed filaments at 30 MPa (average cell length 19±2 µm; 6 cells). Taken together, these results suggest inhibition of cell division in the *dnaT, priA* and *tatC* mutants at 30 MPa. The *rodZ* mutant shows an aberrant spherical morphology at 0.1 MPa, as observed in other studies [40], [41]; these cells remain spherical at 30 MPa (average cell diameter 1.15±0.12 µm at 30 MPa (11 cells) compared to 1.20±0.04 µm at 0.1 MPa (59 cells)). In all cases, the complemented strains showed cell morphology close to that of the parent strain, at both 0.1 MPa and 30 MPa (Figure 3).

### Three Additional Genes are Essential for Growth at 40 MPa

**Figure 4:**
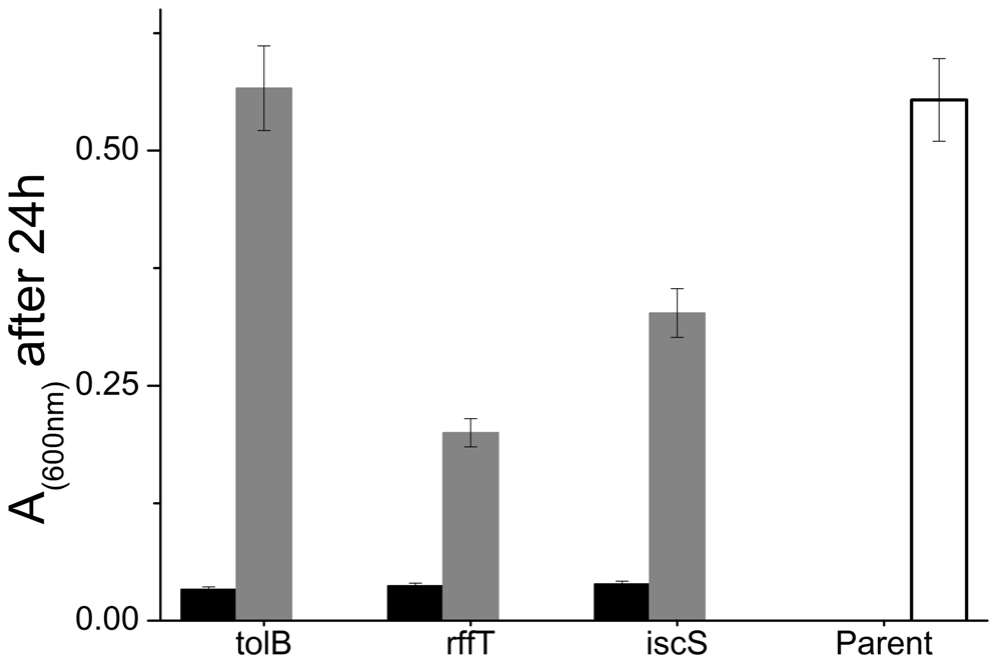
The *tolB, rffT* and *iscS* mutants fail to grow at 40 MPa; growth is restored by complementation. Absorbance at 600 nm is shown for cultures of the mutant and complemented strains after 24 hours of growth at 40 MPa and 37°C. The results are the average of 8 replicate wells on the same plate. As in Figure 2, error bars are computed including variability between replicate plates. From Figure S2, we find the average standard deviation in measured absorbance across replicate plates is 8% of the mean absorbance. We have used this value to compute the error bars in the figure.

A large body of work has shown that different physiological functions become inhibited at different magnitudes of applied pressure [5], [6], [10], [11], [14], [17], [19], [20], [24], [25]. We therefore hypothesized that screening our collection of 82 candidate mutants which had grown least well at 30 MPa, for growth at a higher pressure, might reveal new pressure-sensitive mutants. Indeed, re-screening the candidate mutant set at 40 MPa revealed a further 3 mutants which were able to grow at 30 MPa, but consistently showed little or no growth at 40 MPa (Figure 4). These mutants lacked the genes *tolB, rffT (*also known as *wecF)* and *iscS*. Complementation of these deletion mutants with plasmids carrying the deleted gene resulted in complete or partial restoration of the ability to grow at 40 MPa, confirming the link between genotype and pressure-sensitive phenotype (Figure 4).

**Figure 5:**
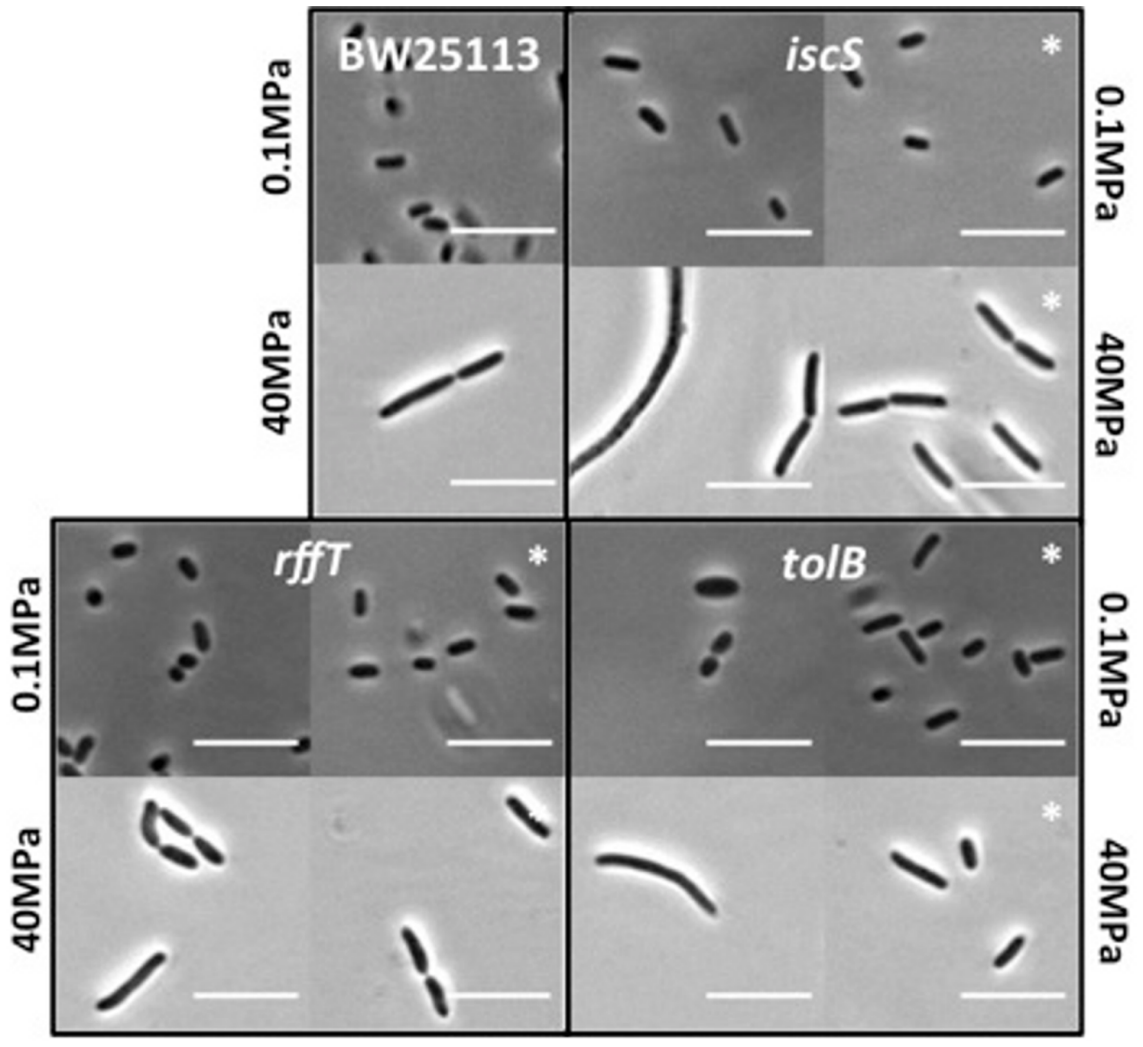
Cell morphology of the three mutants which are sensitive at 40 MPa. Phase contrast microscopy images of the parent strain BW25113 and the *iscS, rffT,* and *tolB* mutants, together with their corresponding complemented strains (indicated by asterisks), after 24 h at 0.1 MPa and 40 MPa. The upper images in each panel show cells grown at 0.1 MPa; the lower images show cells grown at 40 MPa. Left-hand images in each panel show the pressure sensitive mutant; right-hand images show the complemented strain. All scale bars are 10 µm.

Figure 5 shows the cell morphology of the parent strain and the *tolB, rffT* and *iscS* deletion mutants at atmospheric pressure and after 24 h at 40 MPa. Cells of the parent strain BW25113 elongated to approximately the same extent at 40 MPa (average length 2.9±0.2 µm, 24 cells) as at 30 MPa. The *rffT* and *iscS* mutants showed similar morphology to the parent strain, both at atmospheric pressure and at 40 MPa, although a few of the *iscS* cells were filamented at 40 MPa. For *rffT* the average cell lengths were 2.93±0.2 µm (56 cells) at 30 MPa and 1.49±0.02 µm (216 cells) at 0.1 MPa, while for *iscS* they were 5.26±0.85 µm (26 cells) at 30 MPa and 1.41±0.03 µm (109 cells) at 0.1 MPa. The *tolB* mutant showed a slightly “swollen” morphology at atmospheric pressure, but appeared similar to the parent strain at 40 MPa (average cell length was 4.0±0.3 µm (37 cells) at 30 MPa and 1.78±0.06 µm (64 cells) at 0.1 MPa). Interestingly, therefore, it appears that the inability of these 3 deletion mutants to grow at 40 MPa is not manifested in any obvious morphological difference from the parent strain, when imaged at 40 MPa.

We note that seven other deletion mutants, *atpF atpG, dnaK, nusB, tolQ, ybhH* and *ydaS*, also failed to grow at 40 MPa in our rescreening experiments, but were not successfully complemented. These mutants will not be further discussed in this paper.

### Pressure thresholds for Growth

**Figure 6:**
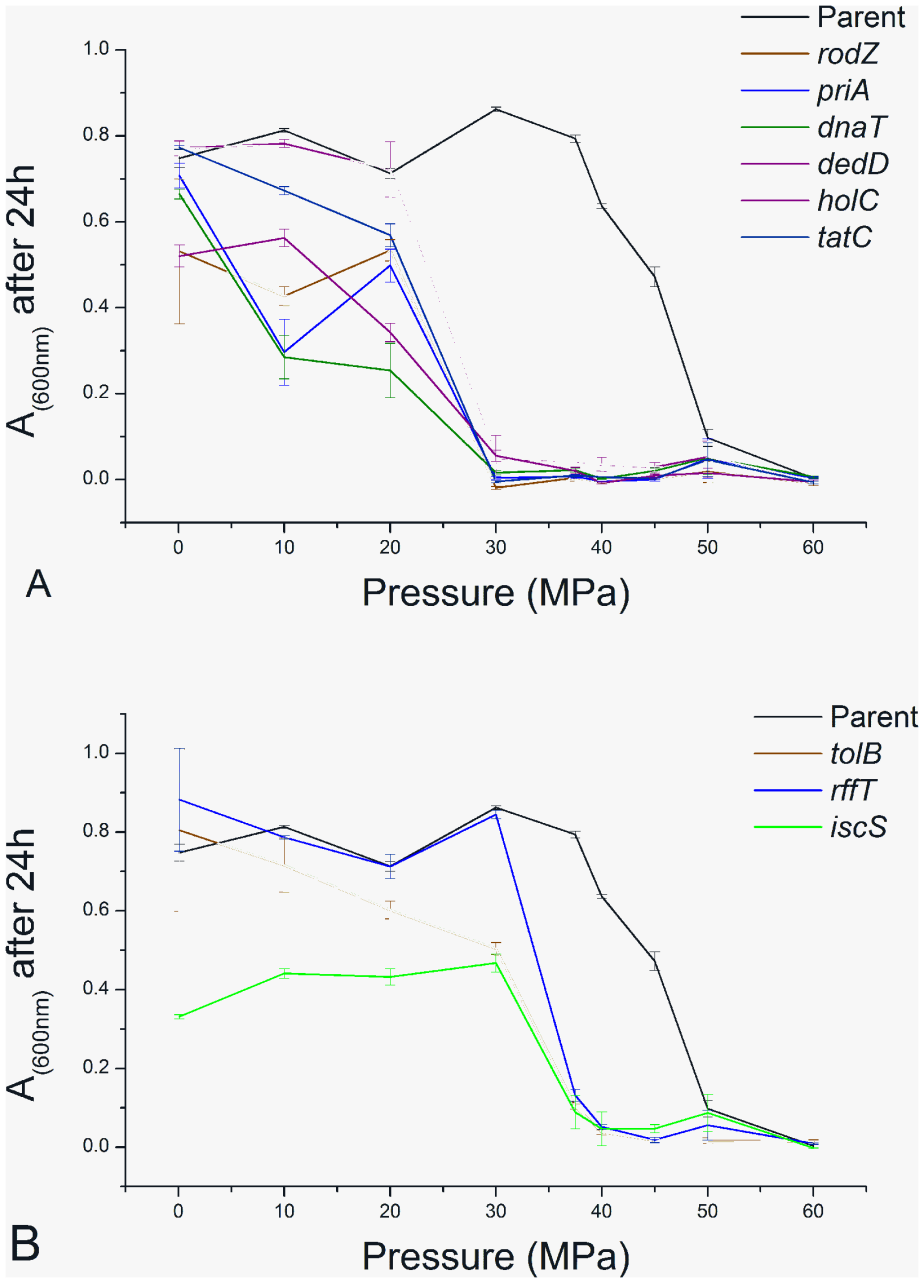
Pressure thresholds for cell growth. Absorbance at 600°C, plotted as a function of pressure. Error bars show the standard error in the mean (SEM) of 4 replicate experiments (16 for the parent strain). Panel A shows results for the parent strain BW25113 and for the 6 mutants identified in our screen as being pressure sensitive at 30 MPa: *rodZ, holC, priA, dnaT, dedD* and *tatC.* Panel B shows results for BW25113 and for the 3 mutants identified in our screen as being pressure sensitive at 40 MPa: *tolB, iscS,* and *rffT.*

To gain further insight into the possible mechanisms by which these gene deletions inhibit growth at pressure, it is interesting to ask whether deletion of a particular gene inhibits growth only above a well-defined pressure threshold, or whether the growth yield of a particular mutant decreases gradually over a wide pressure range. To address this question, we measured the growth of our 9 pressure-sensitive deletion mutants, and the parent strain BW25113, at 10 different pressures in the range 0.1 MPa to 60 MPa (see Methods). For the parent strain BW25113, growth yield drops sharply with pressure over a rather narrow pressure range (30–50 MPa; Figures 6A and 6B). This implies that, below 30 MPa, the cell is apparently able to compensate for the biophysical effects of pressure without a significant decline in growth yield. Several of the mutants identified in our study show similar curves, but shifted to lower pressure – i.e. there is a well-defined pressure threshold for growth, which is reduced compared to that of the parent strain (see for example the curves for *dedD* in Fig. 6A and *rffT* in Fig. 6B). For these mutants the absence of the deleted gene apparently affects the cell’s ability to grow only over a narrow pressure range. In contrast, other mutants show qualitatively different curves, in which growth yield decreases gradually with pressure over a wide range of pressures (see for example the curve for *dnaT* in Fig. 6A). In these cases, the absence of the deleted gene is apparently a limiting factor for growth even at relatively low pressures, and becomes more severe as pressure is increased. This data hints at the possibility of cellular “buffering mechanisms” which can compensate for the biophysical effects of pressure on some physiological processes, but not on others; it will be interesting to investigate this hypothesis further in future work.

## Discussion

Hydrostatic pressure has wide-ranging and important effects on biological molecules and reactions, including changes in lipid membrane structure and the stability of protein complexes. However, it remains unclear how these biophysical effects translate into inhibition of physiological processes *in vivo*, and how the physiology of pressure-tolerant organisms differs from that of those that are pressure-sensitive. The aim of this study was to identify genes that are required for growth of *E. coli* K-12 at pressure, in order to understand better which physiological processes become failure points for growth at pressure in this organism. Our results support existing evidence suggesting that pressure has pleiotropic effects on biophysical processes. In particular, the genes which we identify as being required for growth of *E. coli* BW25113 at pressure are involved in DNA replication, cell division, the cytoskeleton and cell envelope physiology, as we discuss in detail below.

If a particular gene is required for growth at pressure, what does this imply about the underlying molecular mechanisms? We envisage several scenarios in which a gene might be required for growth at high pressure, but not at atmospheric pressure. One possibility is that the deleted gene encodes a protein that stabilizes an essential protein complex, which becomes destabilized at pressure, requiring the stabilizing factor. This may be the case for the RodZ protein, see below. Another possibility is that the protein encoded by the deleted gene forms part of a redundant cellular pathway. At atmospheric pressure, knocking out this pathway has no effect, but at high pressure, alternative pathways fail to operate, and the pathway involving the deleted gene is required for growth. A third possibility is that the deleted protein, while not itself required at pressure, makes available to the cell (e.g. via membrane translocation) another component, which is required at pressure. Importantly, in all these scenarios, the protein product of the deleted gene is not *itself* pressure-sensitive, but rather it compensates, in one way or another, for the pressure-sensitivity of some other cellular components. Thus further biochemical studies, not just of the protein complexes directly identified in this study but also of parallel and connected physiological pathways, would be required to identify the detailed molecular mechanisms leading to pressure sensitivity in these mutants.

### DNA Replication: HolC, DnaT and PriA

*holC* encodes the χ subunit of the multi-component DNA polymerase III holoenzyme - the primary enzyme complex involved in DNA replication in *E. coli*. The χ subunit is a component of the clamp loading (γ) complex, which is involved in the assembly of the polymerase on the DNA: the γ complex assembles the β clamp component of the holoenzyme onto the DNA [42]. The χ subunit interacts directly with single-stranded DNA binding protein (SSB) [42]. Under high salt conditions, omission of χ has been found to lower the efficiency of both clamp loading and DNA polymerase III chain elongation [42]; its effects on chain elongation arise because χ binds to SSB at the lagging strand [43], enhancing processivity. We speculate that similar effects occur under conditions of high pressure: the failure of cells lacking the χ subunit to grow at pressure may be due to failure of clamp loading and/or processivity of the polymerase, due to an increased requirement for the χ-SSB interaction under these conditions. Our observation that *holC* is required for growth above 30 MPa is particularly interesting in the context of existing evidence that the initiation and/or termination of DNA synthesis is inhibited in *E. coli* at 50 MPa [20], and that other proteins involved in DNA replication are required for high pressure growth of the piezophile *P. profundum* [33], [44].

PriA and DnaT are components of the PriA-PriB-DnaT replication restart primosome, which functions to restart DNA replication upon collapse of the DNA replication fork [45], [46]. This protein complex reloads the replisome onto a repaired DNA replication fork or D-loop, following DNA damage. PriA is a helicase that, stimulated by direct interaction with SSB, unwinds branched DNA substrates, providing single-stranded DNA that acts as a loading site for the replicative helicase DnaB. The detailed role of DnaT remains to be established [45], [46]. Previous work has found that mutants in either *dnaT* or *priA* show poor growth and viability, even at atmospheric pressure [47], [48], [49]; this was also the case in our study (Figures 2 and 6A), although a clear pressure-sensitive phenotype was observed. Our results thus point to the restart of stalled or collapsed replication forks as a pressure-sensitive physiological process in *E. coli*. Interestingly, a second pathway to replication restart, involving PriC, is believed to exist in *E. coli*; following scenario 2 above, one may speculate that it might be this pathway that fails at pressure. Consistent with this, mutants in *priB* and *pri*C did not show pressure sensitivity in our study.

### Cytoskeleton: RodZ

RodZ is a recently-discovered inner membrane component of the *E. coli* cytoskeleton [40], [41], [50], [51]. RodZ is essential for maintenance of rod-like cell shape, suggesting that it may play a role in directing the location of peptidoglycan synthesis during growth [40]; mutants lacking *rodZ* are round ([40]; see also Figure 3). RodZ forms helical filaments which colocalise with the MreB actin cytoskeleton; it is believed to interact directly with MreB via its N-terminal cytosolic domain [40] and is required for proper MreB spiral formation. The requirement for RodZ during growth at pressure may reflect pressure-sensitivity of the MreB cytoskeleton – indeed it is well known that eukaryotic actin filaments can be depolymerized by pressure [52], [53], [54]. However, our finding might also be linked to the well-established pressure-induced depolymerization of another cytoskeletal protein, FtsZ [19] - since one of the characteristics of the *rodZ* mutant is its requirement for overexpression of *ftsZ* for good growth on rich media [40].

### Cell division: DedD

DedD is an inner membrane protein that accumulates at the septal ring during cell division, due to its periplasmic C-terminal peptidoglycan-binding SPOR domain [55], [56]. In the absence of FtsN, which is believed to stabilise the septal ring structure, DedD becomes essential [56]. Mutants lacking *dedD* are slightly elongated and tend to form chains, indicating a mild cell division defect [55], [56]. Our finding that DedD is required during growth at pressure suggests that the septal ring structure becomes destabilized under pressure (possibly due to depolymerization of its key component FtsZ [19], or due to other factors). This fits in well with the long-standing existing observation that cell division is inhibited by pressure in *E. coli* [18], [24].

### Cell Envelope Physiology: TolB and RffT

The periplasmic protein TolB is a component of the Tol-Pal protein cell envelope system, which is believed to play a general role in the maintenance of cell envelope integrity [57]–[62] and has also recently been found to be involved in cell envelope constriction at cell division [63]. Tol-Pal consists of inner and outer membrane-associated protein complexes; TolB (with Pal) forms part of the outer membrane-associated complex. The two complexes are connected by an interaction between TolA and Pal, which is driven by the membrane proton motive force [64]. Interestingly, recent work has shown that Tol-Pal accumulates at constriction sites in *E. coli* cells and may be responsible for pulling the outer membrane layer in, as the peptidoglycan and inner membrane layers invaginate during cell constriction [63]. Components of the Tol-Pal system also accumulate at the cell division plane in *Caulobacter crescentus* [65]. The requirement for *tolB* in our experiments may therefore point either to a general sensitivity of the cell envelope, or, more specifically, to envelope constriction during cell division as a weak point in cell physiology during growth at pressure.

The product of the *rffT* gene is also involved in cell envelope physiology, playing a role in cell surface polysaccharide biosynthesis, with implications for outer membrane integrity. *rffT* encodes Fuc4NAc (4-acetamido-4,6-dideoxy-D-galactose) transferase, which is required for the conversion of lipid II to lipid III during the biosynthesis of enterobacterial common antigen (ECA) [66]-[69]. ECA is an outer membrane glycolipid produced by all *Enterobacteriacaeae,* whose biological role is poorly understood [70]. *rffT* mutants show phenotypes suggestive of perturbation of the outer membrane [71]; it has been proposed that this is due to accumulation of lipid II [71]. It therefore appears that the requirement for *rffT* for growth of *E. coli* at pressure arises either from a direct requirement for ECA, or from a general pressure sensitivity of the outer membrane. Pressure is well known to have important biophysical effects on cell membranes [9]: it seems possible, therefore, that the pressure sensitivity of the *rffT* mutant is due to perturbations in its outer membrane which, while not important for growth at atmospheric pressure, become crucial at 30 MPa.

### Protein Export: TatC

The inner membrane protein TatC is a subunit of the TatABCE protein export complex, which transports folded proteins across the inner membrane, powered by the transmembrane proton gradient [72]. TatC is required for the complex’s protein export function [73]. Our observation that TatC is required for growth of *E. coli* above 30 MPa might indicate a requirement for one of its substrates. Intriguingly, mutants which lack a functional Tat system show outer membrane defects [39] and a cell division defect, which causes them to form chains of cells separated by septa (see Figure 3). These defects are caused by failure to translocate two amidases, AmiA and AmiC [74], [75], which are involved in cleavage of the septal peptidoglycan at cell division [74], [76]. One might speculate that the requirement for TatC at pressures about 30 MPa is connected with the pressure-sensitivity of cell division suggested by the other mutants in our study. Although other components of the Tat system were not unambiguously identified in our study, we did observe some pressure sensitivity for both the *tatB* and *tatA* mutants.

### Cellular Supply of Sulphur: IscS

The final gene identified in our study is *iscS*, which we found to be required for growth at 40 MPa. The *iscS* gene encodes the enzyme cysteine desulphurase, which catalyses the supply of sulphur from cysteine, playing a key role in the modification of tRNA by addition of sulphur [77] and in the biosynthesis of iron-sulphur proteins and other sulphur-containing proteins and metabolites [78]. The pressure sensitivity of the *iscS* mutant in our study might therefore either be connected to a requirement for Fe-S clusters (e.g. in the respiratory electron transport chain, which has previously been linked to pressure-sensitivity in *E. coli* [2], [79]), or to the well-documented effects of pressure on translation [21], [80], [81].

### Summary

Our results shed new light on the challenges faced by organisms during growth at high hydrostatic pressure. The 9 genes which we identify as being required for growth of *E. coli* at 30 MPa and 40 MPa point to DNA replication, cell division, the cytoskeleton and cell envelope physiology as potential failure points in cell physiology at pressure.

Our study is not exhaustive: other mutants that are also pressure sensitive may have been missed in our initial screen, or failed to complement in our experiments. It will be interesting to discover whether future experiments reveal other genes involved in these same processes that are required at pressure. For the 9 genes that we did identify, we note that the molecular mechanisms leading to pressure sensitivity upon loss of these genes may well be complex: unravelling the full picture of how and why cells cease to function at pressure calls for biochemical studies, with comparison between pressure-sensitive and pressure-tolerant organisms.

## Supporting Information

Figure S1 **Calibration of the absorbance measurement method.** Solutions of crystal violet of known A_(600 nm)_ were sealed in a microplate as described in Methods (8 replicate wells per A_(600 nm)_ value with 4 medium-only wells as blanks) and pressurized at 30 MPa and 15°C for 60 hours; A_(600 nm)_ was then measured and blanked as described in Methods. The results (shown as symbols with error bars representing SEM) are plotted against the known A_(600 nm)_; the dashed line shows the expected results if the measurement method is perfectly accurate.
(TIF)

Figure S2 **Growth yield measurements are consistent across replicate 96-well plates.** Absorbance at 600 nm was measured across 4 replicate microplates, containing our selected subset of mutants, after 24 h growth at 30 MPa. If the method is reproducible, we would expect a high degree of correlation between A_(600 nm)_ measurements for the same mutant across replicate plates. In the correlation plots shown, each data point represents one mutant. The straight lines show the expected 1∶1 correspondence between measurements in replicate plates; indeed the data is highly reproducible. On average, the standard deviation in measured absorbance for a given mutant, across replicate plates, was 8% of its mean absorbance.
(TIF)

Table S1 **Complete data set for the selected mutant set.** A_(600 nm)_ values at 0.1 MPa and 30 MPa are listed for the selected mutant set, together with their errors (given as standard error in the mean across replicate measurements) and the ratio A_(600 nm)_(30 MPa)/A_(600 nm)_(0.1 MPa). The results are given in order of increasing A_(600 nm)_(30 MPa). Equivalent results for the parent strain BW25113 are given at the end of the table.
(PDF)
