# Supplementary material for: Genes Required for Growth at High Hydrostatic Pressure in Escherichia coli K-12 Identified by Genome-Wide Screening
Source: PLoS One. 2013 Sep 11;8(9):e73995. doi: 10.1371/journal.pone.0073995 (PMC3770679; doi:10.1371/journal.pone.0073995)
Supplement: Table S1 — Complete data set for the selected mutant set. A(600 nm) values at 0.1 MPa and 30 MPa are listed for the selected mutant set, together with their errors (given as standard error in the mean across replicate measurements) and the ratio A(600 nm)(30 MPa)/A(600 nm)(0.1 MPa). The results are given in order of increasing A(600 nm)(30 MPa). Equivalent results for the parent strain BW25113 are given at the end of the table. (PDF) [file pone.0073995.s003.pdf]

Table S1

	A_{600}(0.1MPa)	SEM	A_{600}(30MPa)	SEM	A_{600}(30MPa)/A_{600}(0.1MPa)
priA	**0.71**	**0.03**	**-0.01**	**0.00**	**-0.01**
dnaT	**0.66**	**0.01**	**0.00**	**0.00**	**0.00**
holC	**0.52**	**0.03**	**0.01**	**0.00**	**0.01**
dedD	**0.77**	**0.02**	**0.01**	**0.01**	**0.02**
tatC	**0.77**	**0.00**	**0.02**	**0.01**	**0.03**
rodZ	**0.53**	**0.17**	**0.03**	**0.01**	**0.06**
atpF	0.64	0.02	0.24	0.02	0.37
adhE	0.45	0.03	0.27	0.03	0.61
dnaK	0.51	0.05	0.32	0.03	0.62
atpC	0.45	0.02	0.34	0.03	0.77
tolB	0.80	0.21	0.35	0.03	0.43
gpmI	0.84	0.06	0.35	0.12	0.42
iscS	0.33	0.01	0.36	0.03	1.10
nusB	0.57	0.01	0.38	0.04	0.66
dgkA	0.65	0.01	0.43	0.01	0.67
pta	0.36	0.04	0.44	0.01	1.22
ybcI	0.71	0.01	0.44	0.02	0.62
atpG	0.64	0.03	0.45	0.04	0.71
ybhH	0.71	0.02	0.45	0.04	0.64
tolQ	0.64	0.01	0.46	0.04	0.71
cydD	0.62	0.03	0.48	0.03	0.77
pgi	0.19	0.01	0.48	0.01	2.47
ydaS	0.56	0.01	0.48	0.01	0.86
hfq	0.61	0.02	0.50	0.01	0.81
yeiA	0.69	0.21	0.50	0.01	0.73
yhcB	0.58	0.06	0.52	0.03	0.89
secB	0.72	0.03	0.53	0.02	0.74
moaA	0.56	0.03	0.53	0.00	0.95
rpoN	0.52	0.03	0.56	0.01	1.07
dam	0.56	0.02	0.58	0.03	1.03
dcuC	0.85	0.09	0.58	0.05	0.68
ydaV	0.58	0.01	0.58	0.01	1.00
ymfM	0.74	0.02	0.59	0.00	0.79
modB	0.58	0.01	0.59	0.02	1.01
rffT	0.88	0.13	0.59	0.06	0.67
ybjO	0.70	0.03	0.59	0.02	0.85
moaC	0.56	0.01	0.60	0.03	1.06
moeA	0.57	0.01	0.60	0.02	1.04
moeB	0.57	0.01	0.60	0.01	1.07
selA	0.59	0.01	0.61	0.01	1.03
moaD	0.62	0.01	0.61	0.02	0.98
ynbA	0.58	0.02	0.62	0.02	1.06
moaE	0.61	0.01	0.62	0.02	1.02

yeiH	0.76	0.00	0.62	0.01	0.81
selB	0.62	0.01	0.63	0.01	1.01
lpxL	0.66	0.03	0.64	0.04	0.97
mog	0.59	0.01	0.64	0.01	1.09
yjjP	0.54	0.02	0.65	0.01	1.21
atpB	0.64	0.03	0.66	0.05	1.03
atpE	0.63	0.02	0.66	0.03	1.05
yqeF	0.56	0.01	0.66	0.02	1.18
ddpX	0.72	0.01	0.67	0.01	0.92
yeaV	0.49	0.01	0.69	0.02	1.39
rimM	0.46	0.10	0.69	0.01	1.50
tolR	0.69	0.03	0.70	0.01	1.03
ynjA	0.70	0.01	0.70	0.01	1.01
ytfL	0.71	0.01	0.73	0.01	1.03
ydcQ	0.67	0.01	0.74	0.01	1.11
elaA	0.73	0.03	0.74	0.02	1.02
tas	0.72	0.03	0.75	0.02	1.04
envC	0.73	0.01	0.76	0.03	1.04
gltP	0.56	0.10	0.77	0.04	1.36
ygfZ	0.67	0.01	0.78	0.02	1.17
tatB	0.78	0.01	0.79	0.01	1.00
ackA	0.80	0.05	0.80	0.00	0.99
setB	0.92	0.09	0.81	0.02	0.87
rpoS	0.71	0.01	0.83	0.02	1.18
ybcQ	0.74	0.02	0.84	0.02	1.13
hns	0.70	0.02	0.84	0.02	1.20
yfbU	0.75	0.08	0.84	0.02	1.12
ytfK	0.72	0.02	0.85	0.01	1.17
recD	0.77	0.01	0.85	0.02	1.10
ybbD	0.79	0.01	0.86	0.01	1.09
ydcX	0.75	0.01	0.86	0.01	1.15
asnC	0.82	0.04	0.86	0.00	1.06
yafD	0.76	0.02	0.87	0.01	1.15
ytfH	0.87	0.09	0.88	0.01	1.00
yfcX	0.82	0.00	0.88	0.02	1.08
betI	0.80	0.05	0.88	0.01	1.10
ykgK	0.84	0.00	0.88	0.01	1.05
apaH	0.71	0.01	0.89	0.01	1.24
yehD	0.75	0.02	0.90	0.03	1.19
fliF	0.77	0.02	0.91	0.01	1.19
yniC	0.80	0.01	0.92	0.02	1.14
ydcI	0.75	0.01	0.92	0.01	1.23
wzc	0.77	0.02	0.93	0.02	1.21
Parent	0.75	0.02	0.87	0.02	1.17
